
JOURNAL INFORMATION
==============================
NLM Title Abbreviation: Wirtschaftsdienst
Journal ID: Wirtschaftsdienst
NLM Journal ID: 101086612

Wirtschaftsdienst (Hamburg, Germany : 1949)

ISSN: 0043-6275

ARTICLE INFORMATION
==============================
PMCID: PMC7174810
PMID: 32336796
DOI: 10.1007/s10273-020-2619-1
Article ID: 2619
Article version: 1
Subjects: Kurz Kommentiert

Afrika im Sog des Coronavirus

Africa in the wake of the coronavirus

Kappel Robert robert.kappel@uni-leipzig.de

Universität Leipzig, Institut für Afrikastudien, Beethovenstraße 15, 04107 Leipzig, Deutschland
Electronic publication date: 2020 Apr 22
Print publication date: 2020
Volume: 100
Issue: 4
First page: 229
Last page: 229
Copyright: © Der/die Autor(en) 2020
License: Open Access Dieser Artikel wird unter der Creative Commons Namensnennung 4.0 International Lizenz veröffentlicht, welche die Nutzung, Vervielfältigung, Bearbeitung, Verbreitung und Wiedergabe in jeglichem Medium und Format erlaubt, sofern Sie den/die ursprünglichen Autor(en) und die Quelle ordnungsgemäß nennen, einen Link zur Creative Commons Lizenz beifügen und angeben, ob Änderungen vorgenommen wurden.
Die in diesem Artikel enthaltenen Bilder und sonstiges Drittmaterial unterliegen ebenfalls der genannten Creative Commons Lizenz, sofern sich aus der Abbildungslegende nichts anderes ergibt. Sofern das betreffende Material nicht unter der genannten Creative Commons Lizenz steht und die betreffende Handlung nicht nach gesetzlichen Vorschriften erlaubt ist, ist für die oben aufgeführten Weiterverwendungen des Materials die Einwilligung des jeweiligen Rechteinhabers einzuholen.
Weitere Details zur Lizenz entnehmen Sie bitte der Lizenzinformation auf http://creativecommons.org/licenses/by/4.0/deed.de.
License URL: https://creativecommons.org/licenses/by/4.0/

issue-copyright-statement: © The Author(s) 2020

==============================
DOI: 10.1007/s10273-020-2619-1

COVID-19

### Afrika im Sog des Coronavirus

In fast allen afrikanischen Ländern breitet sich das neuartige Coronavirus aus. Die gemeldeten Fälle steigen, obwohl viele Länder drastische Maßnahmen beschlossen haben. Die Lage in den Krankenhäusern wird dramatisch. Zwar haben die meisten Länder inzwischen Tests, aber die Kapazitäten, schwere Verläufe zu behandeln, sind oft unzureichend: zu wenige Ärzte in zu wenigen Krankenhäusern. Dazu kommt, dass der kontinuierliche Kampf gegen Lassa-Fieber, Gelbfieber, Malaria und HIV-Aids die wenigen Ressourcen bereits stark beansprucht.

Die Wirtschaftskommission der Vereinten Nationen für Afrika (UNECA) geht davon aus, dass sich das wirtschaftliche Wachstum auf unter 2 % verringern könnte und so unter das Bevölkerungswachstum fiele. Ursachen sind eingeschränkte Wirtschaftstätigkeiten und Handelsabschottung. Die weltweite Nachfrage nach Gütern aus Afrika bricht ein. Die Nachfrage nach Rohstoffen und landwirtschaftlichen Produkten sinkt ebenfalls. Da auch die Touristen ausbleiben, sinken Afrikas Exporterlöse. Die Rohstoffpreise sind stark gefallen. Der Economic Vulnerability Index von NKC African Economics weist darauf hin, dass Angola, Gabun, Äthiopien, Ghana, Tunesien, Sambia und Kenia am anfälligsten für Schuldenkrisen sind, falls das Wirtschaftswachstum dauerhaft ausbleibt. Die Folgen für rohstoffexportierende Länder sind gravierend: Sie sind aufgrund undifferenzierter Exportstrukturen in hohem Maße durch Preis- und Nachfrageschwankungen verwundbar. Besonders drastisch ist die Situation in Südsudan, Tschad, Angola, Nigeria oder Sambia. Dort ist der Anteil der Rohstoffexporte besonders hoch. Der starke Rückgang reduziert die Devisenreserven. Niedrigere Austauschraten der Rohstoffexporteure führen zu negativen Einkommenseffekten, die die Ersparnisse reduzieren. Viele Länder Afrikas finanzieren sich durch Handelssteuern und Zölle. Der Preisverfall der Rohstoffe verschlechtert so die Fiskalsalden.

Beispiele können veranschaulichen, welche Folgen die globale Krise hat: Kenias Blumen- und Textilproduktion ist fast komplett eingebrochen, weil es keine Nachfrage mehr aus Europa gibt. In Kenia, Tunesien, den Seychellen und Mauritius ist der Tourismus vollkommen zum Erliegen gekommen. Damit bricht eine wesentliche Quelle von Devisen von jetzt auf gleich weg. Südafrikanische Experten befürchten eine Schrumpfung der Wirtschaft um 2 % und eine weiter steigende Arbeitslosigkeit. Auch die Währungen werden getroffen – der südafrikanische Rand, der nigerianische Naira und der sambische Kwacha verlieren gegenüber dem US-Dollar deutlich an Wert. Auslandsdirektinvestitionen könnten laut Welthandels- und Entwicklungskonferenz (UNCTAD) um 15 % sinken. Die Geldtransfers von Migranten zurück in die Heimat reduzieren sich, weil Arbeitsplätze in Europa, den USA oder den Golfstaaten verloren gehen. Subsahara-Afrika steht vor einem Zahlungsbilanzdefizit von mindestens 100 Mrd. US-$. Mit Südafrika hat das wirtschaftlich bedeutendste Land des Kontinents bereits drastische Maßnahmen zur Eindämmung des Coronavirus und ein Sofortprogramm für die Wirtschaft beschlossen: Stützungsmaßnahmen für Handel und Industrie sowie kleine und mittlere Unternehmen. In vielen anderen Ländern werden ebenfalls Maßnahmen ergriffen, aber die Mittel sind begrenzt. Es ist zu früh zu sagen, wie die Corona-Pandemie in Afrika besonders wirksam eingedämmt werden kann. Hilfe von außen ist trotzdem dringend nötig. Es kommt darauf an, dass die internationalen Organisationen gemeinsam handeln, um zu verhindern, dass auf dem afrikanischen Kontinent eine neue Armutskrise entsteht!

Drei Maßnahmenpakete sind dafür nötig: Erstens müssen öffentliche, private und philanthropische Mittel mobilisiert werden, um Gesundheitssysteme und lokale Produktionen zu stärken. Priorität muss hier die Gesundheitsversorgung haben. Zweitens müssen die Staats- und Regierungschefs der G20 offene Handelskorridore schaffen, um insbesondere Arzneimittel und andere Gesundheitsgüter zu handeln. Darüber hinaus müssen die G20 die Gesundheitsinfrastruktur unterstützen. Dies wird die Länder in die Lage versetzen, sich so weit wie möglich auf die Prävention zu konzentrieren. Die G20 sollten sofort 100 Mrd. US-$ bereitstellen, um unmittelbare Maßnahmen zu ermöglichen. Drittens muss der Internationale Währungsfonds (IWF) mit neuen Sonderziehungsrechten auf die hohe Kapitalflucht aus Afrika reagieren. Mehr als 20 afrikanische Länder haben sich an den IWF gewandt, um Unterstützung zu erhalten. Es muss sicher sein, dass öffentliche Ausgaben zur Eindämmung der Gesundheitskrise nicht durch Finanzkrisen gefährdet werden!

Was als Gesundheitskrise begann, ist schon heute eine globale Wirtschaftskrise. Es besteht die Gefahr, dass Afrika im Sog des Coronavirus alleine gelassen wird. Ohne die nötigen Mittel, die Krise wirksam zu bekämpfen, wird der zu zahlende menschliche und ökonomische Preis verheerend hoch sein. Es ist daher essenziell, dass die reichen Länder erkennen, wie sehr ein taumelndes Afrika auch die eigene wirtschaftliche und soziale Krise verstärkt.

Robert Kappel
Universität Leipzig
robert.kappel@uni-leipzig.de

© Der/die Autor(en) 2020. Open Access: Dieser Artikel wird unter der Creative Commons Namensnennung 4.0 International Lizenz (https://creativecommons.org/licenses/by/4.0/deed.de) veröffentlicht.

Open Access wird durch die ZBW – Leibniz-Informationszentrum Wirtschaft gefördert.
